# Buffalo Medical College

**Published:** 1850-11

**Authors:** 


*Buffalo Medical College.*—The Regular Session of this Institution commences on the 6th inst. About 70 students have been in attendance during the preliminary term.

